# An Asymmetric Genetic Signal Associated with Mechanosensory Expansion in Cave-Adapted Fish

**DOI:** 10.3390/sym12121951

**Published:** 2020-11-26

**Authors:** Amanda K. Powers, Tyler E. Boggs, Joshua B. Gross

**Affiliations:** 1 Department of Genetics, Blavatnik Institute at Harvard Medical School, Boston, MA 02138, USA; 2 Department of Biological Sciences, University of Cincinnati, Cincinnati, OH 45227, USA;

**Keywords:** craniofacial, intramembranous, neuromast, lateral line, QTL analysis

## Abstract

A key challenge in contemporary biology is connecting genotypic variation to phenotypic diversity. Quantitative genetics provides a powerful technique for identifying regions of the genome that covary with phenotypic variation. Here, we present a quantitative trait loci (QTL) analysis of a natural freshwater fish system, *Astyanax mexicanus*, that harbors two morphs corresponding to a cave and surface fish. Following their divergence ~500 Kya, cavefish have adapted to the extreme pressures of the subterranean biome. As a consequence, cavefish have lost numerous features, but evolved gains for a variety of constructive features including behavior. Prior work found that sensory tissues (neuromasts) present in the “eye orbit” region of the skull associate with sensitivity to vibrations in water. This augmented sensation is believed to facilitate foraging behavior in the complete darkness of a cave, and may impact on evolved lateral swimming preference. To this point, however, it has remained unclear how morphological variation integrates with behavioral variation through heritable factors. Using a QTL approach, we discovered the genetic architecture of neuromasts present in the eye orbit region, demonstrating that this feature is under genetic control. Interestingly, linked loci were asymmetric-signals were detected using only data collected from the right, but not left, side of the face. This finding may explain enhanced sensitivity and/or feedback of water movements mediating a lateral swimming preference. The locus we discovered based on neuromast position maps near established QTL for eye size and a facial bone morphology, raising the intriguing possibility that eye loss, sensory expansion, and the cranial skeleton may be integrated for evolving adaptive behaviors. Thus, this work will further our understanding of the functional consequence of key loci that influence the evolutionary origin of changes impacting morphology, behavior, and adaptation.

## 1. Introduction

The blind Mexican tetra is an emerging model system for examining the genetic and cellular basis for cranial asymmetry [1–3]. This freshwater fish species, *Astyanax mexicanus*, includes two morphotypes inhabiting distinct biomes. Cave morphs are restricted to the complete darkness of limestone caves in the Sierra de El Abra region of Northeastern Mexico. As a consequence of life in complete darkness for several hundred thousand years, cave morphs have lost their eyes and pigmentation, but gained several features enabling adaption to this extreme environment [4,5]. Closely-related surface morphs are found broadly distributed throughout the watershed surrounding these caves and, in contrast to cave morphs, demonstrate features typical of teleost fish with normal vision and pigmentation. Surface morphs also have largely symmetric characteristics across the left-right axis with little variation [6]. In contrast, cave-dwelling morphs have evolved extreme cranial asymmetries. This asymmetry is manifested in, at least, three principal features. First, cavefish demonstrate a “bend” in their skulls along the antero-posterior axis that is most often biased to the left [7]. Second, the dermal bones that encircle the eye orbit are frequently fused (synostosed) in an irregular pattern across the left-right axis [6,8,9]. Finally, the largest bone of the circumorbital complex, the third suborbital (SO3, synonymous with infraorbital) bone, is commonly fragmented in an irregular architecture across the lateral cranial complex [8–11].

Despite our knowledge of the developmental origin of asymmetric features in cavefish, an adaptive explanation for these asymmetries remains largely unknown. Specifically, it is unclear how morphological asymmetry may influence behavioral differences in the blind cave morphs. One clue may be a form of directional bias in swimming patterns. Prior research demonstrated that cave-adapted morphs swim in a counterclockwise orientation when sampling novel objects. However, this swimming pattern diminishes once the object is no longer novel [12]. Zebrafish use right-sided vision preferentially for novel objects [13]. Social stimuli are preferentially viewed with left-sided vision [14]. This suggests that asymmetric behavioral phenotypes evolving in cavefish may represent a transfer of the lateral sensory regulation of vision demonstrated in sighted teleosts [12].

**Figure 1.**
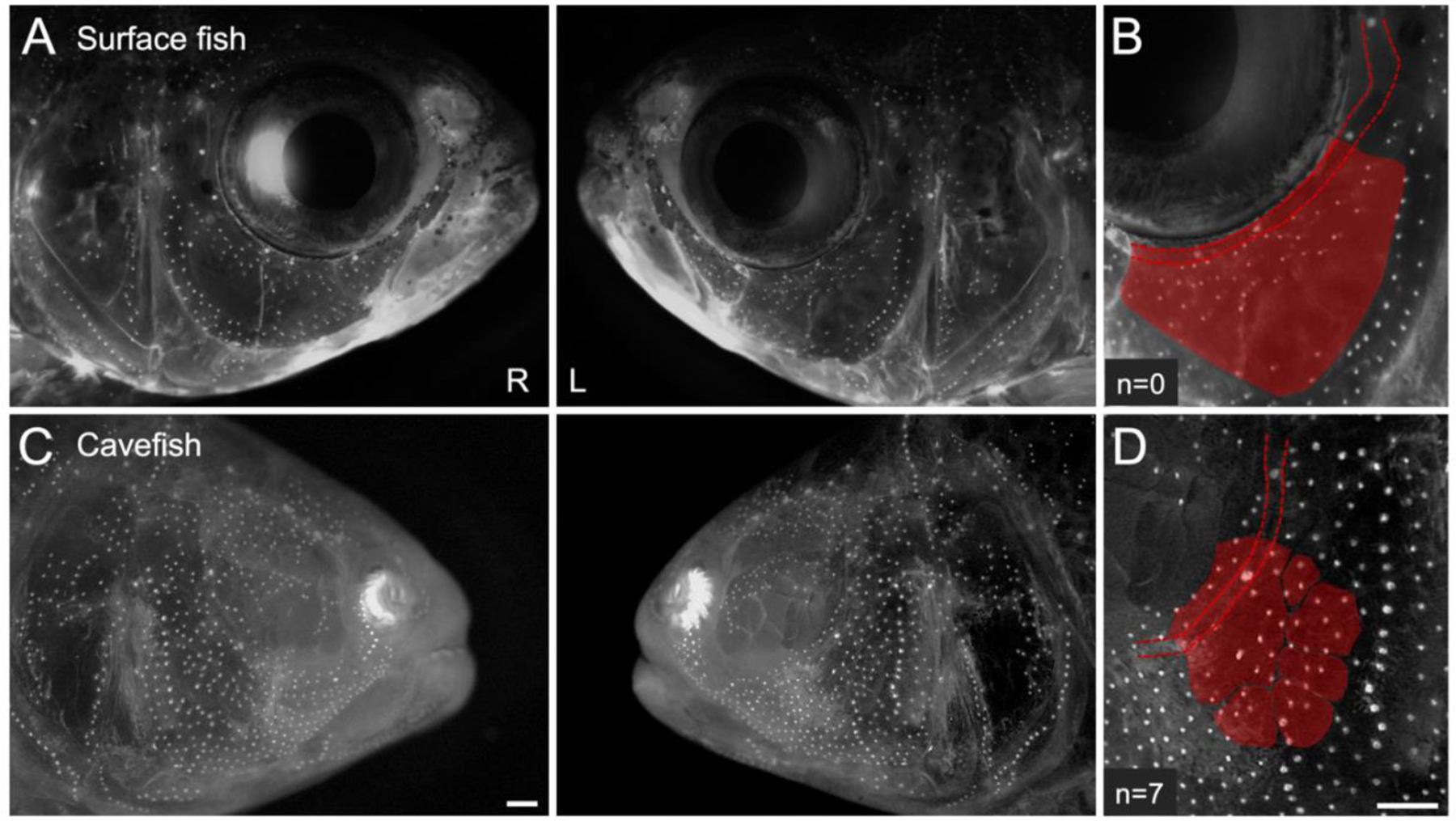
Cavefish exhibit superficial neuromasts within the empty eye orbit. Surface fish have large, round eyes encircled by semi-ossified canals (**A**). The infraorbital canal runs through the suborbital bones (SO3 shown in red) and in surface fish (**B**), acts as a boundary between the eye orbit, dermal bones, and the superficial neuromasts that reside on the surface of the bones. In contrast, eye regression in cavefish causes the orbit to collapse by disrupting the positioning of facial bones, canals, and neuromasts (**C**). Furthermore, the size of the eye orbit and position of facial bones and neuromasts are asymmetric across the midline of the face. In cavefish, both the SO3 bone and associated superficial neuromasts shift into the empty eye orbit (**D**). White scale bars are set to 1 mm.

Touch sensitivity in cavefish is mediated by the lateral line, which is a sensory system comprised of mechanosensory neuromasts (Figure 1A–C) providing positional information in response to water movement. Yoshizawa et al. (2010) discovered that sensory neuromasts mediate the “vibration attraction” response in cave morphs [15]. Neuromasts distributed within the “eye orbit” region of the face (Figure 1D) seem to be particularly important in mediating the attraction to vibrating movement in the water. It is unclear, however, whether morphological asymmetry in cranial neuromasts influence behavioral asymmetry in cave morphs. If a relationship exists between asymmetric neuromast distribution and lateral swimming preference, one may expect to find fixed genetic differences between morphotypes.

Previously, Yoshizawa et al. (2012) discovered that the number of eye orbit neuromasts, eye size, and vibration attraction behavior are under genetic control and colocalize within the same genomic region, but did not report evidence of laterality [16]. Here, we examined the presence of genetic signals associated with asymmetrical morphologies in neuromast distribution in the orbital region. Accordingly, we performed a quantitative trait loci (QTL) analysis of numerical variation in sensory neuromasts in the eye-orbit region on the right and left sides of the face. We discovered the presence of an asymmetric genetic signal, associated with neuromasts on the right side, but not the left side. This finding is consistent with putative genetic changes that may govern variation in a crucial morphological trait shown to be associated with variation in behavioral differences associated with life in the total darkness of a cave.

## 2. Materials and Methods

### 2.1. Genetic Mapping Pedigree

Phenotypic and genotypic data were collected from an F_2_ hybrid mapping pedigree (n = 129) bred from a cross of full sibling F_1_ hybrids derived from a mating between a male surface fish and female cavefish (Pachón cave population). Male surface-dwelling morphs were derived from live-caught specimens collected at the Arroyo Sarco in the Río Sabinas and Río Valles drainages near Ciudad Valles (22°00′10.3″ N 99°00′17.5″ W) in San Luís Potosi, Mexico. Pachón cavefish were derived from live-caught specimens collected from the Pachón cave near El Pachón, Tamaulipas (22°36′32.5″ N 99°03′13.1″ W) in the Sierra de El Abra region of Northeastern Mexico. All specimens used in this study were generously provided to us by Dr. Richard Borowsky of New York University. Fish were reared in individual 1 L tanks as described in Powers et al. (2018) [11]. Genomic DNA extraction, genetic marker generation (genotyping-by-sequencing technology), and linkage map construction were completed previously by Carlson et al. (2015) [17]. In total, 3003 genetic markers were evaluated over 29 linkage groups.

### 2.2. Quantitative Measurements

Fish were evaluated for the distribution of superficial cranial neuromasts. Accordingly, each specimen was immersed in a solution of 2-(4-(dimethylamino)styryl)-N-ethylpyridinium iodide (DASPEI; Sigma Aldrich D3418) diluted in fresh, sterile system water [16]. Under fluorescence, each specimen was imaged under a Leica stereoscope at 7.81× magnification (Leica M205FA, v3.8, Leica Microsystems, Buffalo Grove, IL, USA) using the texas red (TXR) filter (596 nm) to excite DASPEI molecules. Next, each individual was scored for eye size (area in mm^2^) and the number of superficial neuromasts residing within the anatomical “eye orbit” (EO) region of the cranium (Figure 1B,D) for all pedigree members. To assess variation across the midline of the face, each specimen was scored for the presence and number of superficial neuromasts in the left-sided and right-sided EO region. Herein, we defined this anatomical region as the dorsolateral region of face that extends beyond the infraorbital canal not inclusive of neuromasts embedded in the canal. Typically, surface fish do not harbor neuromasts in this region since it is occupied by a structural eye. This resulted in a non-normal distribution across hybrid individuals (Figure S1). Eye size was measured using the polygon selection tool in the open-source Fiji application [18]. Pixels were converted to millimeters (mm) in Microsoft excel (v.16.41). Pairwise t-tests were conducted using StatPlus:mac LE (v7.3.31).

### 2.3. Quantitative Trait Loci (QTL) Analysis

Using scoring information from our experimental pedigree, we performed a series of QTL scans in r/QTL [19]. Each trait was screened using each of three mapping algorithms: maximum regression (MR), expectation maximum (EM), and Haley-Knott (HK) calculations using functions reported in Gross et al. (2014, Figure 2 and Figure S1) [8]. Once completed, the logarithm of the odds (LOD) score was collated across each algorithm and the left-sided and right-sided results were compared. A statistical significance threshold for LOD scores was determined using permutation testing (1000 iterations) for each trait. To determine the polarity of identified signals, we evaluated the phenotypic effect of associated QTL for each marker that rose above the significance threshold.

### 2.4. GO Term Analyses and Literature Review

To evaluate the genes within a critical interval surrounding our linked genomic marker, we evaluated the draft surface fish genome, ASTYANAX 2.0 (NCBI). For each candidate marker, we assessed all genes within an interval spanning ~3 Mb on the distal end of Chromosome 20. This interval included approximately 88 genes, in which all were examined for a potential role in sensory expansion surrounding the circumorbital bone complex. Accordingly, all genes were subjected to manual identification of gene ontology (GO) terms, focused on the following biological process terms: bone, cell migration, craniofacial, facial, nerve, neuromast, vibration, and vision. Additionally, each gene was individually examined for references in the literature to physiological processes implicated in bone and sensory development, maintenance, and function.

## 3. Results

### 3.1. Eye Orbit Superficial Neuromast Number is an Asymmetric Genetic Trait

As a result of eye loss, the cavefish cranium has undergone extensive changes impacting the shape of the orbit as well as the number and position of cranial bones and superficial neuromasts. In the absence of a large eye to maintain the round shape of the orbit, the circumorbital bones and associated canals collapse into the empty space. In particular, the cavefish SO3 bone extends past the infraorbital canal into the orbit and with it, the superficial neuromasts expand and migrate to the dorsal-most boundary of the bone (i.e., EO neuromasts, Figure 1C,D). Because both bone and neuromast traits are asymmetric [20], we evaluated the number of EO neuromasts on both sides of the face in F_2_ hybrids. Within the mapping pedigree, approximately 40% of individuals exhibited surface fish-like large eyes with an absence of EO neuromasts (54/129 on the left and 52/129 on the right sides of the face). For the other hybrids, the number of EO neuromasts ranged from 1–11, resulting in a skewed distribution (Figure S1).

**Figure 2.**
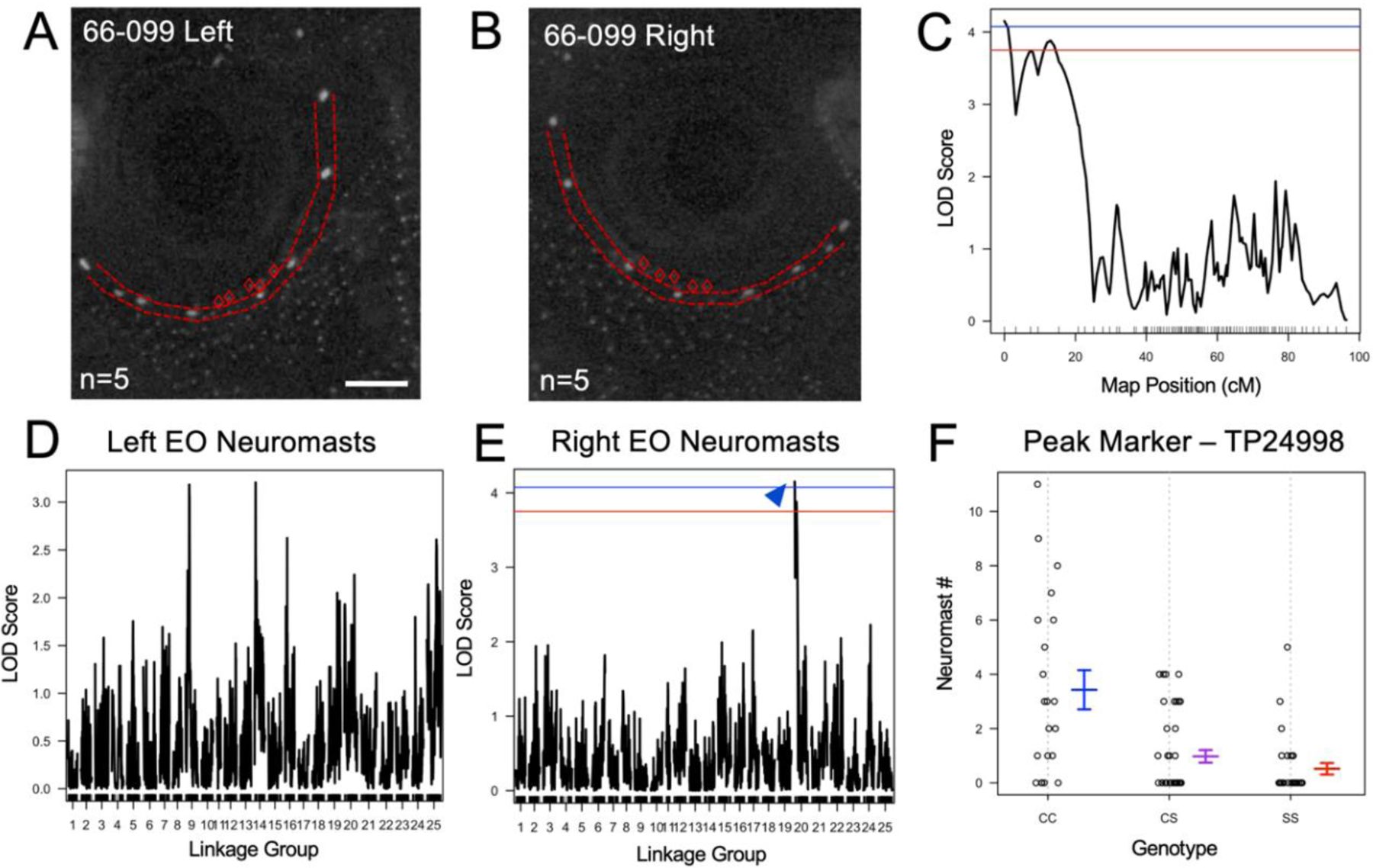
The number of eye orbit neuromasts is an asymmetric genetic trait. A representative F_2_ hybrid individual (66–099) exhibits reduced eyes and five eye orbit (EO) neuromasts on both the left (**A**) and right (**B**) sides of the face. There was no significant genetic signal found for the number of EO neuromasts on the left side of the face (**D**). For the right side of the face, however, a quantitative trait loci (QTL) peak rose above the significance thresholds of *p* < 0.1 (red) and *p* < 0.05 (blue) on Linkage Group 20 (**C**,**E**). For the peak marker (TP24998), the homozygous cavefish (CC) genotype is associated with an increase in the number of EO neuromasts (**F**). The heterozygous (CS) and homozygous surface fish (SS) genotypes are associated with a decrease in the number of EO neuromasts. White scale bar set at 1 mm.

We found that, although within individuals, there is often a difference in the number of EO neuromasts across the midline of the face. Across individuals within the F_2_ pedigree, there is no significant difference between the number on the right vs. the left side (pairwise, *t*-test, *p* = 0.42, Figure 2A,B). There is also no significant difference in eye size between the right and the left sides (pairwise *t*-test, *p* = 0.68). Previous reports of QTL for eye size [21] and neuromast number [16] were scored either on one side of the face or averaged from bilateral scores. The SO3 bone was of particular interest because we previously discovered an asymmetric genetic association with the number of SO3 bony elements (SO3 fragmentation) and suborbital bone fusion [8]. We discovered a QTL signal for the number of EO neuromasts only on the right side of the face. A significant genetic association was found on linkage group (LG) 20 with a logarithm of odds (LOD) score of 4.15 (*p* < 0.05) at the 0 centimorgan (cM) position (Figure 2C,E). Furthermore, the phenotypic effect at the peak genetic marker (TP24998) showed an association between the homozygous cavefish genotype and an increase in the number of EO neuromasts (Figure 2F). When the number of EO neuromasts were scored for the left side of the face, we did not recover a significant genetic association (Figure 2D). Therefore, the number of EO neuromasts, like previously reported QTL for facial bone metrics [8], is an asymmetric genetic trait.

Since the presence of an eye impacts whether neuromasts are displaced into the eye orbit, we performed QTL analysis on eye size. A significant genetic signal for eye size (orbit area) colocalizes with the right side EO neuromast number at the locus on LG 20 (LOD = 5.306, *p* < 0.05, Figure S2). Yoshizawa et al. (2012) also reported an overlap between eye size and EO neuromast QTL [16]. By comparing marker positions from previous QTL studies, we found that the QTL for right side EO neuromasts also colocalizes with eye size [8,21], melanophore count, peduncle depth, SO3 width, and anal fin ray number [21].

### 3.2. Genomic Analyses Reveal Candidate Genes Associated with Eye Orbit Neuromast Number

**Figure 3.**
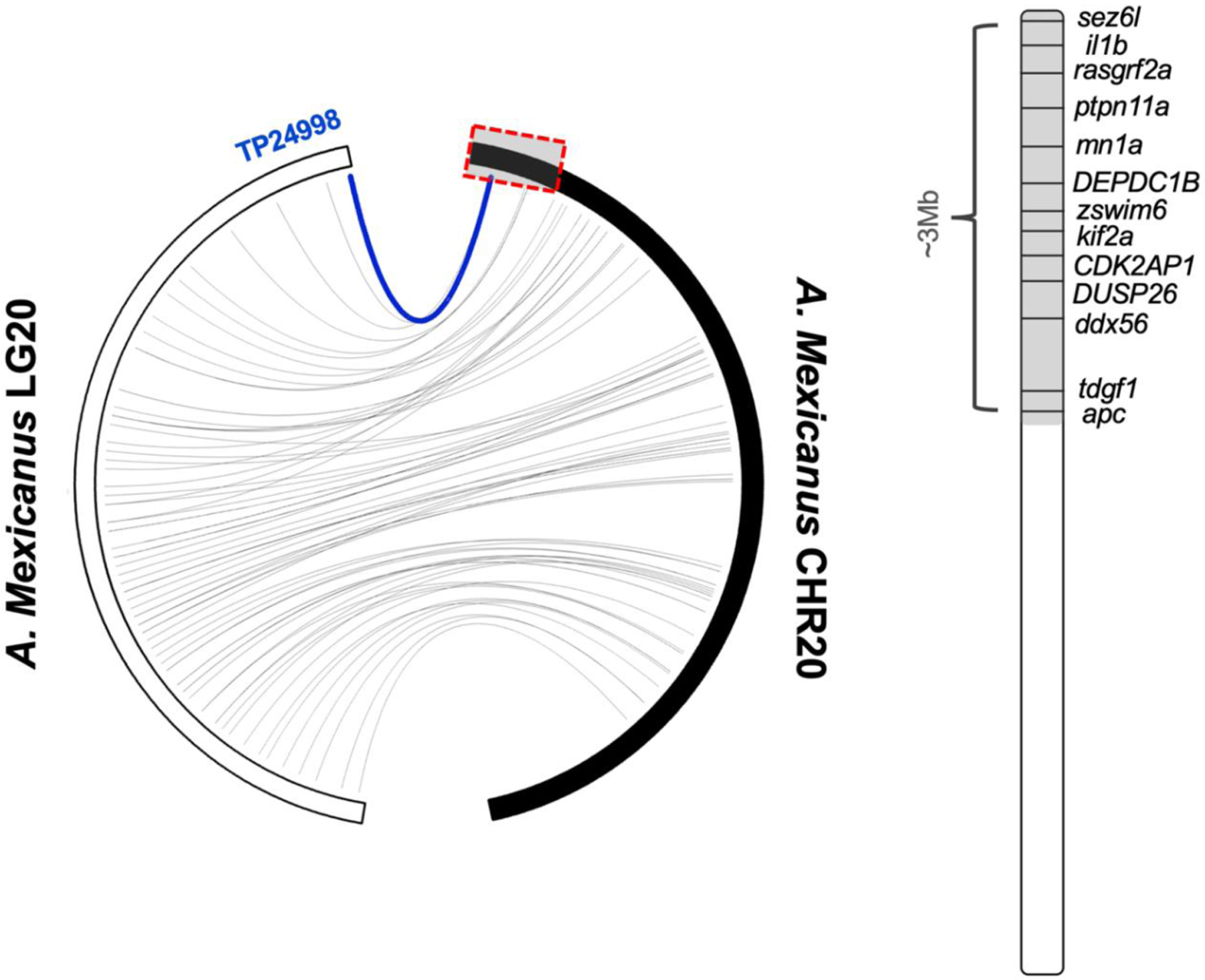
The critical region of Linkage Group 20 is syntenic with the distal end of *A. mexicanus* Chromosome 20. The top maker associated with right side eye orbit (EO) neuromast number (TP24998 in blue) resides at approximately ~0cM on Linkage Group 20 (LG). The syntenic region on *A. mexicanus* Chromosome 20 includes ~90 genes within a ~3Mb region. A candidate gene list of 13 genes was generated based on Gene Ontology (GO) terms.

The sequence from the top hit genetic marker was BLASTed against the *A. mexicanus* 2.0 genome in order to estimate a genomic interval associated with a significant QTL. The peak marker, TP24998, maps to the 914,700 Kb region of Chr. 20 (Figure 3) with an e-value of 4 × 10^−28^ and 100% alignment. The marker did not have a significant e-value associated with the *A. mexicanus* 1.0.2 genome (e-value = 0.73). Approximately 90 genes within a ~3 Mb (1–1.5 Mb on either side of position 20:914700) syntenic region on the distal end of Chr. 20 were subjected to GO term analysis. We discovered 13 candidate genes with biological process GO terms associated with the nervous system or eye development, cell migration, and facial morphogenesis (Table 1).

## 4. Discussion

Life in total darkness is associated with the evolution of asymmetry. For instance, in some cavefish populations, eye loss early in embryogenesis regresses in a lateralized manner across the left-right axis. Prior work in our system also revealed a number of genetic signals associated with craniofacial morphologies that appear to affect the size, structure, and fusion of a variety of intramembranous bones near the orbit [8,21]. Many of the QTL discovered from this work were only identified with phenotypic information collected from one side of the face. This genetic asymmetry is consistent with the discovery of a lateral cranial bend in cavefish, which is not present in surface fish. One possibility is that asymmetric cranial shape changes are integrated alongside neuromast sensitivity. Given that dermal bone shape can covary with superficial neuromast patterns in zebrafish [22], we reasoned that a functional relationship may exist between the lateral line and cranial skeleton.

Our finding that an asymmetric genetic signal exists for neuromast presence within the eye orbit is significant for at least three reasons. First, this finding supports previous reports that neuromast positions are under genetic control [16]. Second, an asymmetric QTL for EO neuromasts provides a plausible link between cranial morphology, lateral line sensitivity, and behavior. Third, the position of this QTL overlaps with the positions of established QTL for both eye size and orbital bone shape. The associated orbital bone (SO3) is particularly densely populated with superficial neuromasts. The presence of QTL for three different traits may indicate a shared genetic basis, or regulatory structure, for one or more genes within the same interval. We feel it is unlikely that the same gene, or genetic lesion, mediates vision loss, bone morphology, neuromast position, and behavior. However, this finding may indicate that certain processes (such as loss of a structural eye) are associated with morphogenetic alterations in cavefish that favor increased sensitivity to water movement. For instance, the loss of a structural eye may facilitate expansion of the dorsal SO3 bone into the orbit. In cavefish, the presence of EO neuromasts appears to be limited to areas occupied by intramembranous bone. It is intriguing to hypothesize that behavioral changes may have evolved as a consequence of eye loss through integration between the sensory and skeletal systems. In order to determine if there is a relationship between these traits, first we must characterize the gene or genes underlying cave-associated traits.

Toward that end, we investigated the genomic region wherein our QTL aligns and discovered 13 candidate genes associated with biological processes relevant to the eye orbit neuromast trait. The genes *il1b* [23,24], *DEPDC1B* [25,26], *zswim6* [27], *kif2a* [28], and *tdgf1* [29] have known roles in cell migration, which is a process likely involved in the displacement of neuromast positioning. Furthermore, the genes *ptpn11a* [30,31], *mn1a* [32,33], and *CDK2AP1* [34,35] are reportedly involved in ossification and facial morphogenesis, which could link sensory and skeletal asymmetry in cavefish. Finally, the genes *sez6l* [36], *rasgrf2a* [37], and *ddx56* [38], *DUSP26* [39–41] and *apc* [42–44] have been implicated in neural tissue and eye development. Additional work is necessary to identify the causative gene (and lesion) that mediates this trait and perform functional assays to determine how two different structures (neuromasts and bone) are implicated in the evolution of adaptive behaviors.

## 5. Conclusions

Cavefish have evolved unusual behavioral patterns (vibration attraction, lateral swimming preference) as a consequence of life in total darkness. These behavioral patterns likely reflect changes in peripheral reception as well as the central and peripheral nervous systems. Peripheral touch reception is mediated, in part, by mechanosensory neuromasts sensitive to water movement patterns. Here, we demonstrate an asymmetric genetic signal associated with the presence of mechanosensory neuromasts in the eye orbit region, which is an anatomical position important for behavioral evolution in cavefish. This genetic signal overlaps with previously discovered eye size loci, raising the intriguing possibility that the same genomic region controls both reductions of vision and gains in mechanosensation. Future work will further explore putative lesions within the coding and promoter regions of candidate genes and identify the functional impact of these changes on morphology, behavior, and adaptation.

## Supplementary Material

symmetry-12-01951-s001

## Figures and Tables

**Table 1.** Candidate genes for eye orbit (EO) neuromast number based on location and Gene Ontology (GO).

Candidate Gene	Location	Associated Biological Processes	References
*sez6l*	20: 652–15,946	Synapse maturation, Neuronal cell body	Beck et al. 2015
*Mb*	20: 169,678–180,459	Cell migration, Synaptic transmission, Neurogenesis	Bourdiec et al. 2013; Brun et al. 2018
*rasgrfla*	20: 291,929–310,752	Long-term synaptic potentiation	Ma et al. 2014
*ptpnlla*	20: 514,486–527,912	Positive regulation of ossification, Facial morphogenesis, Intestinal epithelial cell migration	Araki et al. 2004; Yang et al. 2013
*mnla*	20: 871,152–874,436	Ossification	Xiang et al. 2013; Miyake et al. 2020
*DEPDC1B*	20: 1,330,269–1,337,096	Cell migration	Huang et al. 2017; Zhang et al. 2020
*zswim6*	20: 1,383,303–1,480,986	Neuron migration	Smith et al. 2014
*kifla*	20: 1,517,483–1,535,540	INervous system development, Cell migration	Watanabe et al. 2015
*CDKlAPl*	20: 1,630,465–1,634,317	Facial morphogenesis	Kim et al. 2009; Zolochevaka & Figueiredo, 2011
*DUSP16*	20: 1,749,854–1,751,288	Retina layer formation, Neuron development	Rissone et al. 2019; Kim et al. 2020; Warren et al. 2020
*ddx56*	20: 2,009,592–2,018,474	Positive regulation of neuron projection development	Romero-Carvajal, 2015
*tdgfi*	20: 2,718,586–2,726,122	Neural tube closure, Neural plate anterior/posterior regionalization, Positive regulation of cell migration	Aoto et al. 2009
*apc*	20: 2,781,236–2,817,251	Retina development in camera-type eye, Skeletal system development, Cell migration, Eye photoreceptor cell differentiation	Monaghan et al. 2001; Shao et al. 2016; Tang et al. 2019

## References

[1] Atukorallaya DSA; Ratnayake RK Shaping the craniofacial skeleton of Mexican cavefish (Astyanax mexicanus); Role of Osteoblast and Osteoclast. FASEB J. 2018, 32, 776.15.

[2] Gross JB; Powers AK A natural animal model system of craniofacial anomalies: The blind Mexican cavefish. Anat. Rec 2020, 303, 24–29. 10.1002/ar.23998 PMC6606388 30365238

[3] McGaugh SE; Kowalko J; Duboue ER; Lewis P; Franz-Odendaal TA; Rohner N; Gross JB; Keene AC Dark world rises: The emergence of cavefish as a model for the study of evolution, development, behavior, and disease. J. Exp. Zool. Part B Mol. Dev. Evol 2020. 10.1002/jez.b.22978 PMC7736471 32638529

[4] Culver DC Cave Life: Evolution and Ecology; Harvard University Press: Cambridge, MA, USA, 1982.

[5] Jeffery WR Cavefish as a Model System in Evolutionary Developmental Biology. Dev. Biol 2001, 231, 1–12. 11180948 10.1006/dbio.2000.0121

[6] Gross JB; Powers AK The Evolution of the Cavefish Craniofacial Complex: Biology and Evolution of the Mexican Cavefish, 1st ed; Elsevier: San Diego, CA, USA, 2015.

[7] Powers AK; Davis EM; Kaplan SA; Gross JB Cranial asymmetry arises later in the life history of the blind Mexican cavefish, Astyanax mexicanus. PLoS ONE 2017, 12, e0177419. 28486546 10.1371/journal.pone.0177419 PMC5423691

[8] Gross JB; Krutzler AJ; Carlson BM Complex Craniofacial Changes in Blind Cave-Dwelling Fish Are Mediated by Genetically Symmetric and Asymmetric Loci. Genetics 2014, 196, 1303–1319. 24496009 10.1534/genetics.114.161661 PMC3982692

[9] Powers AK; Boggs TE; Gross JB Canal neuromast position prefigures developmental patterning of the suborbital bone series in Astyanax cave- and surface-dwelling fish. Dev. Biol 2018, 441, 252–261. 29630866 10.1016/j.ydbio.2018.04.001 PMC6119090

[10] Yamamoto Y; Espinasa L; Stock DW; Jeffery WR Development and evolution of craniofacial patterning is mediated by eye-dependent and -independent processes in the cavefish Astyanax. Evol. Dev 2003, 5, 435–446. 12950623 10.1046/j.1525-142x.2003.03050.x

[11] Powers AK; Kaplan SA; Boggs TE; Gross JB Facial bone fragmentation in blind cavefish arises through two unusual ossification processes. Sci. Rep 2018, 8, 7015. 29725043 10.1038/s41598-018-25107-2 PMC5934472

[12] Burt de Perera T; Braithwaite VA Laterality in a non-visual sensory modality—The lateral line of fish. Curr. Biol 2005, 15, 241–242. 10.1016/j.cub.2005.03.035 15823520

[13] Miklóski A; Andrew RJ; Savage H Behavioural lateralization of the tetrapod type in the zebrafish (*Brachydanio rerio*). Physiol. Behav 1998, 63, 127–135. 10.1016/s0031-9384(97)00418-6 9402625

[14] Sovrano VA; Rainoldi C; Bisazza A; Vallortigara G Roots of brain specializations: Preferential left-eye use during mirror-image inspection in six species of teleost fish. Behav. Brain Res. 1999, 106, 175–180. 10595433 10.1016/s0166-4328(99)00105-9

[15] Yoshizawa M; Gorički Š; Soares D; Jeffery WR Evolution of a Behavioral Shift Mediated by Superficial Neuromasts Helps Cavefish Find Food in Darkness. Curr. Biol 2010, 20, 1631–1636. 20705469 10.1016/j.cub.2010.07.017 PMC2946428

[16] Yoshizawa M; Yamamoto Y; O’Quin KE; Jeffery WR Evolution of an adaptive behavior and its sensory receptors promotes eye regression in blind cavefish. BMC Biol. 2012, 10, 108. 23270452 10.1186/1741-7007-10-108 PMC3565949

[17] Carlson BM; Onusko SW; Gross JB A High-Density Linkage Map for *Astyanax mexicanus* Using Genotyping-by-Sequencing Technology. G3 Genes Genomes Genet. 2015, 5, 241–251. 10.1534/g3.114.015438 PMC4321032 25520037

[18] Schindelin J; Arganda-Carreras I; Frise E; Kaynig V; Longair M; Pietzsch T; Preibisch S; Rueden C; Saalfeld S; Schmid B; Fiji: An open-source platform for biological-image analysis. Nat. Methods 2012, 9, 676–682. 22743772 10.1038/nmeth.2019 PMC3855844

[19] Broman KW; Wu H; Sen Ś; Churchill GA R/qtl: QTL mapping in experimental crosses. Bioinformatics 2003, 19, 889–890. 12724300 10.1093/bioinformatics/btg112

[20] Gross JB; Gangidine A; Powers AK Asymmetric Facial Bone Fragmentation Mirrors Asymmetric Distribution of Cranial Neuromasts in Blind Mexican Cavefish. Symmetry 2016, 8, 118. 28078105 10.3390/sym8110118 PMC5221661

[21] Protas M; Tabansky I; Conrad M; Gross JB; Vidal O; Tabin CJ; Borowsky R Multi-trait evolution in a cave fish, *Astyanax mexicanus*. Evol. Dev 2008, 10, 196–209. 18315813 10.1111/j.1525-142X.2008.00227.x

[22] Wada H; Ghysen A; Satou C; Higashijima S-I; Kawakami K; Hamaguchi S; Sakaizumi M Dermal morphogenesis controls lateral line patterning during postembryonic development of teleost fish. Dev. Biol 2010, 340, 583–594. 20171200 10.1016/j.ydbio.2010.02.017

[23] Bourdiec A; Calvo É; Rao CV; Akoum A Transcriptome Analysis Reveals New Insights into the Modulation of Endometrial Stromal Cell Receptive Phenotype by Embryo-Derived Signals Interleukin-1 and Human Chorionic Gonadotropin: Possible Involvement in Early Embryo Implantation. PLoS ONE 2013, 8, e64829. 23717664 10.1371/journal.pone.0064829 PMC3661534

[24] Brun NR; Koch BEV; Varela M; Peijnenburg WJGM; Spaink HP; Vijver MG Nanoparticles induce dermal and intestinal innate immune system responses in zebrafish embryos. Environ. Sci. Nano 2018, 5, 904–916.

[25] Huang L; Chen K; Cai Z-P; Chen F-C; Shen H; Zhao-Peng C; Yang S-J; Chen X-B; Tang G-X; Lin H DEPDC1 promotes cell proliferation and tumor growth via activation of E2F signaling in prostate cancer. Biochem. Biophys. Res. Commun 2017, 490, 707–712. 28634077 10.1016/j.bbrc.2017.06.105

[26] Zhang S; Shi W; Hu W; Ma D; Yan D; Yu K; Zhang G; Cao Y; Wu J; Jiang C; DEP Domain-Containing Protein 1B (DEPDC1B) Promotes Migration and Invasion in Pancreatic Cancer through the Rac1/PAK1-LIMK1-Cofilin1 Signaling Pathway. Onco Targets Ther. 2020, 13, 1481–1496. 32110046 10.2147/OTT.S229055 PMC7035893

[27] Smith JD; Hing AV; Clarke CM; Johnson NM; Perez FA; Park SS; Horst JA; Mecham B; Maves L; Nickerson DA; Exome Sequencing Identifies a Recurrent De Novo *ZSWIM6* Mutation Associated with Acromelic Frontonasal Dysostosis. Am. J. Hum. Genet 2014, 95, 235–240. 25105228 10.1016/j.ajhg.2014.07.008 PMC4129399

[28] Watanabe T; Kakeno M; Matsui T; Sugiyama I; Arimura N; Matsuzawa K; Shirahige A; Ishidate F; Nishioka T; Taya S; TTBK2 with EB1/3 regulates microtubule dynamics in migrating cells through KIF2A phosphorylation. J. Cell Biol. 2015, 210, 737–751. 26323690 10.1083/jcb.201412075 PMC4555816

[29] Aoto K; Shikata Y; Imai H; Matsumaru D; Tokunaga T; Shioda S; Yamada G; Motoyama J Mouse Shh is required for prechordal plate maintenance during brain and craniofacial morphogenesis. Dev. Biol 2009, 327, 106–120. 19103193 10.1016/j.ydbio.2008.11.022

[30] Araki T; Mohi MG; A Ismat F; Bronson RT; Williams IR; Kutok JL; Yang W; I Pao L; Gilliland DG; Epstein JA; Mouse model of Noonan syndrome reveals cell type- and gene dosage-dependent effects of *Ptpn11* mutation. Nat. Med 2004, 10, 849–857. 15273746 10.1038/nm1084

[31] Yang W; Wang J; Moore DC; Liang H; Dooner MS; Wu Q; Terek R; Chen Q; Ehrlich MG; Quesenberry PJ; Ptpn11 deletion in a novel progenitor causes metachondromatosis by inducing hedgehog signalling. Nature 2013, 499, 491–495. 23863940 10.1038/nature12396 PMC4148013

[32] Xiang L; Li M; Liu Y; Cen J; Chen Z; Zhen X; Xie X; Cao X; Gu W The clinical characteristics and prognostic significance of MN1 gene and MN1-associated microRNA expression in adult patients with de novo acute myeloid leukemia. Ann. Hematol 2013, 92, 1063–1069. 23515710 10.1007/s00277-013-1729-x

[33] Miyake N; Takahashi H; Nakamura K; Isidor B; Hiraki Y; Koshimizu E; Shiina M; Sasaki K; Suzuki H; Abe R; Gain-of-Function MN1 Truncation Variants Cause a Recognizable Syndrome with Craniofacial and Brain Abnormalities. Am. J. Hum. Genet 2020, 106, 13–25. 31839203 10.1016/j.ajhg.2019.11.011 PMC7042485

[34] Kim Y; McBride J; Kimlin L; Pae E-K; Deshpande A; Wong DT Targeted Inactivation of p12Cdk2ap1, CDK2 Associating Protein 1, Leads to Early Embryonic Lethality. PLoS ONE 2009, 4, e4518. 19229340 10.1371/journal.pone.0004518 PMC2641017

[35] Zolochevska O; Figueiredo ML Cell-cycle regulators cdk2ap1 and bicalutamide suppress malignant biological interactions between prostate cancer and bone cells. Prostate 2011, 71, 353–367. 20812223 10.1002/pros.21249

[36] Beck M; Peterson JF; McConnell J; McGuire M; Asato M; Losee JE; Surti U; Madan-Khetarpal S; Rajkovic A; Yatsenko SA Craniofacial abnormalities and developmental delay in two families with overlapping 22q12.1 microdeletions involving theMN1gene. Am. J. Med. Genet. Part A 2015, 167, 1047–1053. 10.1002/ajmg.a.36839 25810350

[37] Ma X; Espana-Serrano L; Kim W-J; Purayil HT; Nie Z; Daaka Y βArrestin1 Regulates the Guanine Nucleotide Exchange Factor RasGRF2 Expression and the Small GTPase Rac-mediated Formation of Membrane Protrusion and Cell Motility. J. Biol. Chem 2014, 289, 13638–13650. 24692549 10.1074/jbc.M113.511360 PMC4036368

[38] Romero-Carvajal MA Regeneration of Sensory Hair Cells and Progenitor Self-Renewal Require Localized Interactions between the Notch and Wnt Signaling Pathways Ph.D. Thesis, The University of Utah, Salt Lake City, UT, USA, 2015. 10.1016/j.devcel.2015.05.025 PMC4557215 26190147

[39] Rissone A; Jimenez E; Bishop K; Carrington B; Slevin C; Wincovitch SM; Sood R; Candotti F; Burgess SM A model for reticular dysgenesis shows impaired sensory organ development and hair cell regeneration linked to cellular stress. Dis. Model. Mech 2019, 12, dmm040170. 10.1242/dmm.040170 PMC6955229 31727854

[40] Kim T-Y; Neupane S; Aryal YP; Lee E-S; Kim J-Y; Suh J-Y; Lee Y; Sohn W-J; An S-Y; Ha J-H; Implications of specific gene expression patterns in enamel knot in tooth development. Int. J. Oral Biol. 2020, 45, 25–31.

[41] Warren WC; Boggs TE; Borowsky R; Carlson BM; Ferrufino E; Gross JB; Hillier L; Hu Z; Keene AC; Kenzior A; A chromosome level genome of *Astyanax mexicanus* surface fish for comparing population-specific genetic differences contributing to trait evolution. bioRxiv 2020. 10.1038/s41467-021-21733-z PMC7933363 33664263

[42] Monaghan H; Bubb VJ; Sirimujalin R; Millward-Sadler SJ; Salter DM *Adenomatous polyposis coli* (APC), beta-catenin, and cadherin are expressed in human bone and cartilage. Histopathology 2001, 39, 611–619. 11903580 10.1046/j.1365-2559.2001.01287.x

[43] Shao R; Liu J; Yan G; Zhang J; Han Y; Guo J; Xu Z; Yuan Z; Liu J; Malumbres M; Cdh1 regulates craniofacial development via APC-dependent ubiquitination and activation of Goosecoid. Cell Res. 2016, 26, 699–712. 27126000 10.1038/cr.2016.51 PMC4897181

[44] Tang D; He Y; Li W; Li H Wnt/β-catenin interacts with the FGF pathway to promote proliferation and regenerative cell proliferation in the zebrafish lateral line neuromast. Exp. Mol. Med 2019, 51, 1–16. 10.1038/s12276-019-0247-x PMC6533250 31123246

