# Supplementary material for: An Asymmetric Genetic Signal Associated with Mechanosensory Expansion in Cave-Adapted Fish
Source: Symmetry (Basel). Author manuscript; Available in PMC 2021 Feb 19. (PMC7894647; doi:10.3390/sym12121951)
Supplement: symmetry-12-01951-s001 [file NIHMS1671285-supplement-symmetry-12-01951-s001.pdf]

Supplementary

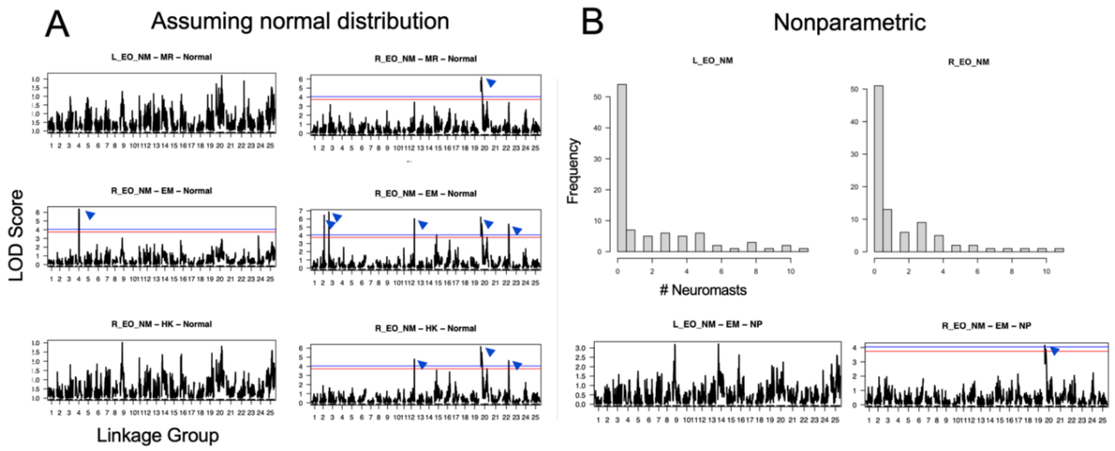

Figure S1. Different statistical methods used during quantitative trait loci (QTL) analysis reveal different genetic associations. We employed three QTL mapping methods: maximum regression (MR), expectation maximum (EM), and Haley-Knott (HK) under normal (**A**) and nonparametric (**B**) conditions. Peaks that rose above the significance threshold ($p < 0.05$, LOD = 4.01) are denoted with a red arrow. QTL analysis was performed for the number of eye orbital (EO) on the left and right sides of the face.

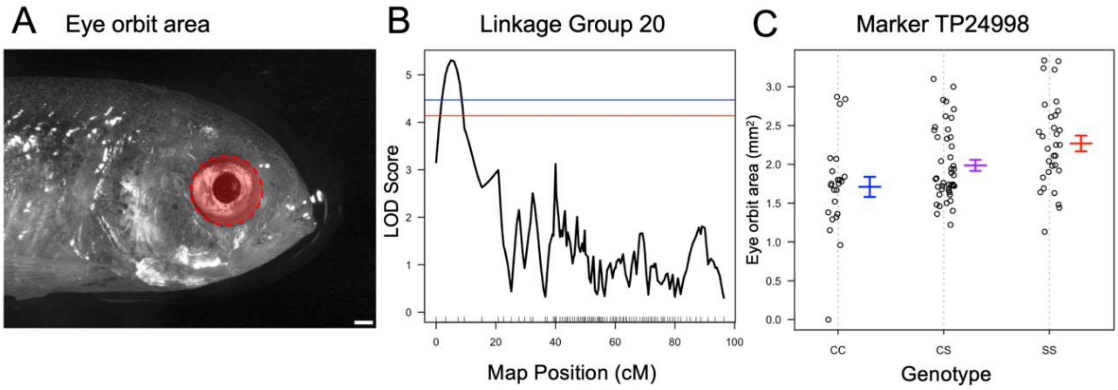

Figure S2. Eye orbit area and EO neuromast number QTL co-localize to the same locus. Eye orbit size was measured in mm^2 (**A**) and was significantly associated with LG 20 (LOD 5.306, $p < 0.05$) (**B**). A larger eye orbit is associated with the homozygous surface fish genotype (SS) (**C**). White scale bar indicates 1 mm.
